# Supplementary material for: A single-cell RNA-sequencing training and analysis suite using the Galaxy framework
Source: Gigascience. 2020 Oct 20;9(10):giaa102. doi: 10.1093/gigascience/giaa102 (PMC7574357; doi:10.1093/gigascience/giaa102)
Supplement: giaa102_Response_to_Reviewer_Comments_Original_Submission [file giaa102_response_to_reviewer_comments_original_submission.pdf]

Author's Response To Reviewer Comments

Close

____

REVIEWER COMMENTS

____

Table of Contents

____

1. Reviewer #1
.. 1. Types of sequencing
.. 2. Alternate Platforms
.. 3. Cell Ranger
.. 4. Other Pseudotime Packages
.. 5. Galaxy: Free or Freemium?
.. 6. Size of scRNA-seq analyses
2. Reviewer #2
.. 1. Small typos
.. 2. Suggestions

Dear Sir/Madam,

Thank you for the reviews and the positive feedback. We have taken your comments and suggestions into much consideration, and here we address them below one by one:

1 Reviewer #1
=============

1.1 Types of sequencing
~~~~~~~~~~~~~~~~~~~~~~~~~

In the abstract, it would be better if the author could emphasize this Galaxy workflow is only used to 3'-end transcript sequencing. Based on the description of the manuscript, it seems not work on full-transcript sequencing data.

The custom pre-processing workflow that we offer should actually work on any kind of sequencing data, since the mapping is normally performed at the genome level (via STAR), and the quantification can be performed at either the gene, transcript or exon level. This has been used to find rare transcripts using SMART-Seq2 FASTQ data. I have changed the abstract to mention that we can perform quantification on both tagged and full-length protocols.

1.2 Alternate Platforms
~~~~~~~~~~~~~~~~~~~~~~~~~

In the abstract, 10x Genomics has been mentioned several

times, it gives the impression that Galaxy is used to analyze 10x scRNA-seq data. But in the Conclusion of Abstract, the paper mentioned that it works to both 10x and alternative derived datasets. Could you be specific on alternative derived datasets? Does that mean other platform data? The paper addresses the analyses in other platform sequencing.

I have changed the abstract conclusion to "alternative platforms" instead of "alternately derived datasets", but I do not wish to name specific protocols because then it will give the false impression that the analyses are restricted to only these, however I have now directly named some of these protocols in the "Flexible Pre-processing" section.

1.3 Cell Ranger
~~~~~~~~~~~~~~~

On page 5 of PDF file under 10x Analysis Software category, it describes that 10x Genomics provided software. The software in the paper should be Cell Ranger if I understand it correctly. I could not see Cell Ranger in Galaxy workflow, but found the description in the Methods, which seems STARsolo was used to replace Cell Ranger. I checked on STARsolo paper, which has 95% association with Cell Ranger. A lot of people may prefer to run Cell Ranger for 10x data, it would be great to have Cell Ranger as an option in Galaxy pipeline.

We have considered Cell Ranger before, but there are fundamental software and licensing issues that prevent us from including it in Galaxy. Namely, though Cell Ranger is free to download, the non-standard [software license agreement] is very restrictive in the distribution of it (in particular Section 4.1). When STARsolo was released shortly after, with a standard open-source license as well as a ten-fold speed increase to reproduce the same analysis, we gladly went with it.

[software license agreement]

1.4 Other Pseudotime Packages
~~~~~~~~~~~~~~~~~~~~~~~~~~~~~

On page 9 of PDF file under Pseudotime Techniques, Is PAGA the only technique used in trajectory analysis for Scanpy workflow? There are about 70 methods that can be used to do trajectory analysis, PAGA is not that popular to be selected, is that possible to consider adding other one or two methods to Galaxy?

We also have the FateID and StemID packages from Grün et al, and the PAGA package was mentioned because it was integrated into the upstream ScanPy source. All future updates from ScanPy will be automatically included into the Galaxy wrapper in this manner, and the community also has plans to extend the current set of single cell tools further.

1.5 Galaxy: Free or Freemium?

~~~~~~~~~~~~~~~~~~~~~~~~~~~~~~~~

On page 9 of PDF file under Discussion with Cloud-based Analysis. It's a little bit not clear to me. If a user creates Galaxy account, but he/she doesn't have cloud account, is he/she able to run scRNA-seq analysis using Galaxy with cloud computation for free? Galaxy should be a very good user-friendly tool to allow people to use. Due to tremendous number of cells for one study, it may require the large memory and CPU to support the system to run the analysis. If Galaxy is able to solve the computation problem, it would be helpful for the researchers to analyze the data using Galaxy. Please clarify if Galaxy can do the cloud computation, but the academic users also need to pay for the usage or account.

The Galaxy framework is free and open source software, and can be deployed across many different compute resources such as your own local machine or through cloud services. There are over 7000 tools available that can be deployed across all Galaxy instances, though instances typically contain a curated subset of the total (> 2500). The Galaxy service provided by these instances can vary between deployment strategies, and so it is possible to have commercial services which run Galaxy, e.g. through Amazon Web Services.

However the Galaxy service, as provided by the European Galaxy server in this paper, is deployed on compute resources which are funded by ELIXIR and de.NBI, which both support a European-wide publicly-funded effort to bring scientific computing infrastructure to scientists and users alike.

The European Galaxy server currently sports 2 PB of storage, 8000 processing cores and 40 TB of RAM, which it puts to use through a smart scheduling system. The server is free, there is no payment plan or hidden fees, and the project runs without adverts. The European Galaxy server aims to stay free for as long as funding exist.

An anonymous user can freely run an analysis without worrying about cost. If they were a registered user (also free and no hidden costs), they would be able to reap the benefits of being able to log in from multiple machines as well as being able to use extended storage.

1.6 Size of scRNA-seq analyses
~~~~~~~~~~~~~~~~~~~~~~~~~~~~~~~~

The similar question to 5 in Availability of supporting data and materials. UseGalaxy.eu server seems the main server for users to run the analysis, it would be helpful to provide more information, for example, what's the capability for this server in running scRNA-seq data? How many max cells can be run at once in this server?

That's a hard question to answer, because the scheduling of these jobs try to ensure that all concurrent users recieve a fair amount of compute time on the server. Jobs are also grouped by their size, where large jobs such as STAR are dispatched into larger compute nodes, and smaller jobs such as ScanPy are dispatched into smaller nodes, and so the maximum processing capacity is decided by the size and current load of the node. On the rare occasion that compute power is low, the jobs are simply queued and then started later, which is not a visible concern should you run jobs overnight.

I myself have run several large (~ 100) STAR jobs in parallel, with each FASTQ file having reads for ~3000 cells. I have not registered any noticeable lag in processing, and I can say the same for smaller jobs too. The most number of cells I have ever processed downstream in one sitting is 15,000 cells and I don't recall any issues. I am also certain that much larger datasets have been processed by other users.

2 Reviewer #2

2.1 Small typos

Other Approaches. The pre-processing workflow for these -> Other Approaches. The pre-processing workflows for these

A missing parenthesis in the "Downstream Workflows" section of the manuscript (two of which are shown in Figure 2, each -> (two of which are shown in Figure 2), each

Both are now fixed, thank you.

2.2 Suggestions

In the "Pre-processing Workflows" section of the manuscript I suggest you make the name and availability of each published workflow clearer, perhaps by introducing it in the appropriate paragraph's title. e.g. Quantification with STARsolo -> Quantification with "10x STARsolo" workflow, Flexible Pre-processing - > Flexible Pre-processing with "CelSeq2: Single Batch mm10" workflow

I am resistant to the idea of changing the headers, in particular the Flexible Pre-processing headers to directly reference CelSeq2 because then it limits the workflow to just that protocol, when in reality the workflow can be adapted very easily for any kind of protocol. I have referenced the workflow names directly in the text however, in order to better direct users to these workflows. The full list of all workflows can be found on the [single cell web portal].

[single cell web portal]

I suggest you do the same in the "Downstream Workflows" section e.g. Scater-based Quality Control -> Quality Control with "Single-Cell Quality Control with Scater" Workflow (I suggest the same for the rest of the workflows)

I have changed the sections of these headers to "Quality Control with Scater", "Downstream Analysis with the ScanPy Suite", and "Downstream Analysis with the RaceID Suite", since the actual workflow names here do not follow a common naming scheme and are slightly jarring to read.

In the "Downstream Workflows" section of the manuscript you mention that there are five main stages of downstream scRNA-seq analysis but in the following paragraph it is not very clear which of the three workflows contain

them. It becomes quite clear (the 2 after the pre-analysis
workflow) in the next page from the Figure 2. explanation
but I suggest that you mention it during the description
of each workflow. e.g. stage is complete, the full
downstream analysis can be performed -> stage is complete,
the full downstream analysis (comprising the five stages
mentioned above) can be performed

I have made this change, thank you.

Overall this is a very well written paper that verifies
once more Galaxy's huge potential in -omic data analysis
and specifically focuses on sincle cell transcriptomic
data to prove that point.

Thanks once more!

Close
