# Supplementary material for: A single-cell RNA-sequencing training and analysis suite using the Galaxy framework
Source: Gigascience. 2020 Oct 20;9(10):giaa102. doi: 10.1093/gigascience/giaa102 (PMC7574357; doi:10.1093/gigascience/giaa102)
Supplement: giaa102_Reviewer_1_Report_Original_Submission — Li Jia -- 7/7/2020 Reviewed [file giaa102_reviewer_1_report_original_submission.pdf]

Reviewer Report

Title: A Single-cell RNA-seq Training and Analysis Suite using the Galaxy Framework

Version: Original Submission **Date:** 7/7/2020

Reviewer name: Li Jia

Reviewer Comments to Author:

1. In the abstract, it would be better if the author could emphasize this Galaxy workflow is only used to 3'-end transcript sequencing. Based on the description of the manuscript, it seems not work on full-transcript sequencing data.
2. In the abstract, 10x Genomics has been mentioned several times, it gives the impression that Galaxy is used to analyze 10x scRNA-seq data. But in the Conclusion of Abstract, the paper mentioned that it works to both 10x and alternative derived datasets. Could you be specific on alternative derived datasets? Does that mean other platform data? The paper addresses the analyses in other platform sequencing.
3. On page 5 of PDF file under 10x Analysis Software category, it describes that 10x Genomics provided software. The software in the paper should be Cell Ranger if I understand it correctly. I could not see Cell Ranger in Galaxy workflow, but found the description in the Methods, which seems STARsolo was used to replace Cell Ranger. I checked on STARsolo paper, which has 95% association with Cell Ranger. A lot of people may prefer to run Cell Ranger for 10x data, it would be great to have Cell Ranger as an option in Galaxy pipeline.
4. On page 9 of PDF file under Pseudotime Techniques, Is PAGA the only technique used in trajectory analysis for Scanpy workflow? There are about 70 methods that can be used to do trajectory analysis, PAGA is not that popular to be selected, is that possible to consider adding other one or two methods to Galaxy?
5. On page 9 of PDF file under Discussion with Cloud-based Analysis. It's a little bit not clear to me. If a user creates Galaxy account, but he/she doesn't have cloud account, is he/she able to run scRNA-seq analysis using Galaxy with cloud computation for free? Galaxy should be a very good user-friendly tool to allow people to use. Due to tremendous number of cells for one study, it may require the large memory and CPU to support the system to run the analysis. If Galaxy is able to solve the computation problem, it would be helpful for the researchers to analyze the data using Galaxy. Please clarify if Galaxy can do the cloud computation, but the academic users also need to pay for the usage or account.
6. The similar question to 5 in Availability of supporting data and materials. UseGalaxy.eu server seems the main server for users to run the analysis, it would be helpful to provide more information, for example, what's the capability for this server in running scRNA-seq data? How many max cells can be run at once in this server?

Level of Interest

Please indicate how interesting you found the manuscript: Choose an item.

Quality of Written English

Please indicate the quality of language in the manuscript: Choose an item.

Declaration of Competing Interests

Please complete a declaration of competing interests, considering the following questions:

- Have you in the past five years received reimbursements, fees, funding, or salary from an organisation that may in any way gain or lose financially from the publication of this manuscript, either now or in the future?
- Do you hold any stocks or shares in an organisation that may in any way gain or lose financially from the publication of this manuscript, either now or in the future?
- Do you hold or are you currently applying for any patents relating to the content of the manuscript?
- Have you received reimbursements, fees, funding, or salary from an organization that holds or has applied for patents relating to the content of the manuscript?
- Do you have any other financial competing interests?
- Do you have any non-financial competing interests in relation to this paper?

If you can answer no to all of the above, write 'I declare that I have no competing interests' below. If your reply is yes to any, please give details below.

No. I declare that I have no competing interests

I agree to the open peer review policy of the journal. I understand that my name will be included on my report to the authors and, if the manuscript is accepted for publication, my named report including any attachments I upload will be posted on the website along with the authors' responses. I agree for my report to be made available under an Open Access Creative Commons CC-BY license (http://creativecommons.org/licenses/by/4.0/). I understand that any comments which I do not wish to be included in my named report can be included as confidential comments to the editors, which will not be published.

Choose an item.

To further support our reviewers, we have joined with Publons, where you can gain additional credit to further highlight your hard work (see: https://publons.com/journal/530/gigascience). On publication of this paper, your review will be automatically added to Publons, you can then choose whether or not to claim your Publons credit. I understand this statement.

Yes Choose an item.
