# Supplementary material for: A single-cell RNA-sequencing training and analysis suite using the Galaxy framework
Source: Gigascience. 2020 Oct 20;9(10):giaa102. doi: 10.1093/gigascience/giaa102 (PMC7574357; doi:10.1093/gigascience/giaa102)
Supplement: giaa102_Reviewer_2_Report_Original_Submission — Efthymios Ladoukakis, Ph.D -- 7/10/2020 Reviewed [file giaa102_reviewer_2_report_original_submission.pdf]

Reviewer Report

Title: A Single-cell RNA-seq Training and Analysis Suite using the Galaxy Framework

Version: Original Submission **Date:** 7/10/2020

Reviewer name: Efthymios Ladoukakis, Ph.D

Reviewer Comments to Author:

Small typos:
Other Approaches. The pre-processing workflow for these -> Other Approaches. The pre-processing workflows for these
A missing parenthesis in the "Downstream Workflows" section of the manuscript
(two of which are shown in Figure 2, each -> (two of which are shown in Figure 2), each
----
Suggestions:
In the "Pre-processing Workflows" section of the manuscript I suggest you make the name and availability of each published workflow clearer, perhaps by introducing it in the appropriate paragraph's title. e.g.
Quantification with STARsolo -> Quantification with "10x STARsolo" workflow
Flexible Pre-processing - > Flexible Pre-processing with "CelSeq2: Single Batch mm10" workflow
I suggest you do the same in the "Downstream Workflows" section e.g.
Scater-based Quality Control -> Quality Control with "Single-Cell Quality Control with Scater" Workflow
(I suggest the same for the rest of the workflows)
In the "Downstream Workflows" section of the manuscript you mention that there are five main stages of downstream scRNA-seq analysis but in the following paragraph it is not very clear which of the three workflows contain them. It becomes quite clear (the 2 after the pre-analysis workflow) in the next page from the Figure 2. explanation but I suggest that you mention it during the description of each workflow. e.g.
stage is complete, the full downstream analysis can be performed -> stage is complete, the full downstream analysis (comprising the five stages mentioned above) can be performed
Overall this is a very well written paper that verifies once more Galaxy's huge potential in -omic data analysis and specifically focuses on sincle cell transcriptomic data to prove that point.

Level of Interest

Please indicate how interesting you found the manuscript: Choose an item.

Quality of Written English

Please indicate the quality of language in the manuscript: Choose an item.

Declaration of Competing Interests

Please complete a declaration of competing interests, considering the following questions:

- Have you in the past five years received reimbursements, fees, funding, or salary from an organisation that may in any way gain or lose financially from the publication of this manuscript, either now or in the future?
- Do you hold any stocks or shares in an organisation that may in any way gain or lose financially from the publication of this manuscript, either now or in the future?
- Do you hold or are you currently applying for any patents relating to the content of the manuscript?
- Have you received reimbursements, fees, funding, or salary from an organization that holds or has applied for patents relating to the content of the manuscript?
- Do you have any other financial competing interests?
- Do you have any non-financial competing interests in relation to this paper?

If you can answer no to all of the above, write 'I declare that I have no competing interests' below. If your reply is yes to any, please give details below.

I declare that I have no competing interests

I agree to the open peer review policy of the journal. I understand that my name will be included on my report to the authors and, if the manuscript is accepted for publication, my named report including any attachments I upload will be posted on the website along with the authors' responses. I agree for my report to be made available under an Open Access Creative Commons CC-BY license (http://creativecommons.org/licenses/by/4.0/). I understand that any comments which I do not wish to be included in my named report can be included as confidential comments to the editors, which will not be published.

Choose an item.

To further support our reviewers, we have joined with Publons, where you can gain additional credit to further highlight your hard work (see: https://publons.com/journal/530/gigascience). On publication of this paper, your review will be automatically added to Publons, you can then choose whether or not to claim your Publons credit. I understand this statement.

Yes Choose an item.
